# Cognitive implications of white matter microstructural changes in individuals with low heart rate variability: a NODDI study

**DOI:** 10.3389/fneur.2025.1503599

**Published:** 2025-05-09

**Authors:** Shaofan Jiang, Yingzhe Cheng, Ling Luo, Rifeng Jiang, Xiaodong Pan, Yunjing Xue

**Affiliations:** ^1^Department of Radiology, Fujian Medical University Union Hospital, Fuzhou, China; ^2^Department of Neurology, Center for Cognitive Neurology, Fujian Medical University Union Hospital, Fuzhou, China; ^3^Fujian Institute of Geriatrics, Fujian Medical University Union Hospital, Fuzhou, China; ^4^Institute of Clinical Neurology, Fujian Medical University, Fuzhou, China; ^5^Fujian Key Laboratory of Molecular Neurology, Fujian Medical University, Fuzhou, China

**Keywords:** NODDI, cognition, plasma biomarker, MRI, HRV

## Abstract

**Objective:**

This study aimed to investigated the microstructural integrity of WM and the relationship with the cognitive domains and cognition-related plasma biomarkers in low heart rate variability (HRV).

**Methods:**

Our study recruited 44 high HRV and 44 low HRV (Grouping by median of HF). Diffusion Magnetic resonance imaging (MRI) was utilized for the calculation of neurite orientation dispersion and density imaging (NODDI) parameters, and tract-based spatial statistics (TBSS) were employed to explore differential clusters. The fibers covered by these clusters were defined as regions of interest (ROI) for the extraction of NODDI parameter values and the analysis of their correlation with cognitive domains and cognition-related plasma biomarkers.

**Results:**

The TBSS analysis unveiled specific cerebral regions exhibiting disparities within the low HRV group high HRV group. These differences were evident in fractional isotropy (FISO; *p* < 0.05). The extracted values from these ROIs (*p* < 0.05) were mainly manifested in the anterior regions of the brain and corpus callosum. Further analysis showed that the abnormal white matter (WM) showed significant correlations with CDR, RCFT-I, RCFT-D, attention, memory, language, and cognition-related plasma biomarkers to varying degrees.

**Conclusion:**

Patients with low HRV exhibit distinctive patterns of microstructural changes in the WM as revealed by the FISO which indicate a decline in white matter integrity, and the relationship between low HRV-related and worse cognitive performance may be attributed to damage of the frontal-corpus callosum pathways.

## 1 Introduction

The ECG of a healthy human exhibits subtle fluctuations from beat to beat. This phenomenon is known as heart rate variability (HRV), which refers to the variation in the duration of time separating consecutive heart contractions (1). The fluctuations in heart rate are characterized by HRV, a manifestation of the intricate interplay between the sympathetic and parasympathetic branches of the autonomic nervous system (2). HRV has emerged as a significant indicator of overall wellbeing (1, 3).

HRV can be measured by using methods from the time domain and frequency domain (4, 5). The standard time-domain measurements for HRV typically encompass the Standard Deviation of the N-N Intervals (SDNN), the Root Mean Square of the Successive Differences (RMSSD), and the percentage of successive normal sinus RR intervals more than 50 ms (pNN50). In the frequency domain, the key parameters are the total power (TP), high-frequency band (HF; 0.15 to 0.4 Hz), low-frequency band (LF; 0.04 to 0.15 Hz), very-low frequency (VLF; 0.0033 to 0.04 Hz), and ultralow frequencies (ULF; below 0.0033 Hz) (6). SDNN captures the overall variability in the heart rate across the entire recording period, including all the cyclic fluctuations. RMSSD reflects vagal tone. The pNN50 metric is correlated with both RMSSD and the HF, and it also serves as an indicator of the parasympathetic activity (7). ULF reflects overall autonomic activity, including core body temperature, metabolism and the renin-angiotensin system. VLF is an indicator of the combined activity of both the sympathetic and parasympathetic nervous systems, with a notable predominance of sympathetic influence. HF reflects the highest level of efferent parasympathetic (vagal) influence on the sinus node. LF reflects a mix of sympathetic and vagal nerve effects, it shows the influence of sympathetic and parasympathetic nerve branches. A shared aspect of these HRV parameters is their emphasis on autonomic nervous function, an area that is also central to the majority of HRV research studies. Despite a lack of consensus, cumulative studies have shown that lower HRV is reflective of autonomic nervous dysautonomia (8).

HRV is a widely utilized method for evaluating the state of the autonomic nervous system (9). Previous studies have shown that the autonomic nervous system is related to self-regulation in aspects such as cognition, emotion, social and health (10, 11). Therefore, the relationship between HRV and global cognitive performance is emerging as a hot topic of research. Multiple studies have established a connection between the autonomic nervous system and cognitive processes, showing that there is a beneficial association between cognitive performance and HRV (12–14). However, there has been controversy about the relationship between HRV and its connection to the broad cognitive domain. As is widely known, the cognitive domain encompasses a variety of mental functions, including but not limited to the following key areas: attention, memory, language, learning ability and visuospatial function. Hanse et al. (15) and Colzato et al. (16) found that higher HRV was associated with better memory and attention but other studies concluded that the relationship between HRV and memory was not significant (17). Britton et al. (18) enrolled 5,375 middle-aged individuals and found that reduced HRV was not related to cognitive disorders in the middle-aged population. From the studies aforementioned, many of these inconsistencies are probably due to diverse populations studied, inconsistency in HRV measurement or the different components of the cognitive domains included in these studies (4). Consequently, a quantitative evaluation method is needed.

Neuroimaging analysis can provide quantitative information of brain structure. Over the past years, despite the fact that voxel-based morphometry (VBM) and functional magnetic resonance imaging (fMRI) has been used to investigate the association between HRV and global cognitive performance (5, 19, 20), the signal of fMRI is susceptible to the influence of blood flow changes and heart rate (21), and VBM is not sensitive to subtle changes for brain structure. Diffusion imaging methods, such as neurite orientation dispersion and density imaging (NODDI) could be used to measure WM microstructure differences and how they were associated with cognitive impairment (22, 23). The microstructural environments of WM fiber bundle could be measured by neurite density index (NDI), orientation dispersion index (ODI), and fractional isotropy (FISO). Recently, WM is gradually becoming a focus of interest in HRV researches. Yu et al. (24) believes that cardiovascular diseases are risk factors for white matter hyperintensities. One recent study has shown that an inverse association between low nighttime HRV and white matter hyperintensities progression, and provide support for the role of sympathetic overactivity in this relationship (25). Tian et al. (26) also found that reduced RMSSD was associated with white matter hyperintensity. We speculate that changes in the autonomic nervous system may lead to alterations in the WM. Notably, a few studies (27, 28) consider cardiac autonomic dysfunction is associated with cognitive decline, but its underlying pathophysiological mechanisms may be related to white matter lesions in the brain. Therefore, we postulate the following hypothesis: HRV is associated with microstructural changes in WM. Fortunately, NODDI is more sensitive to the density of neurites and the degree of fiber used to describe changes in WM microstructure (29). Unlike DTI, NODDI has multi-compartment biophysical model representing each voxel and three different microstructural environments (30).

As far as we know, scarce studies were available to employ the NODDI model to synthesize assessments of WM damage in low HRV patients, with insufficient attention directed toward elucidating the correlation of cognitive implications in low HRV with combined WM damage. Examining the impact of white matter integrity may help elucidate how HRV impacts brain processes, including memory, attention, language, visuospatial function, and the sensorimotor system. These findings may provide novel insights into the relationship between HRV and cognition.

## 2 Materials and methods

### 2.1 Ethical approval statement

All procedures performed in studies involving human participants followed the ethical standards of the institutional research committee with the 1964 Helsinki Declaration and its later amendments or comparable ethical standards. The study protocol was approved by the Ethics Committee of Fujian Medical University Union Hospital (2021KJT001).

### 2.2 Study subjects

**Figure 1:**
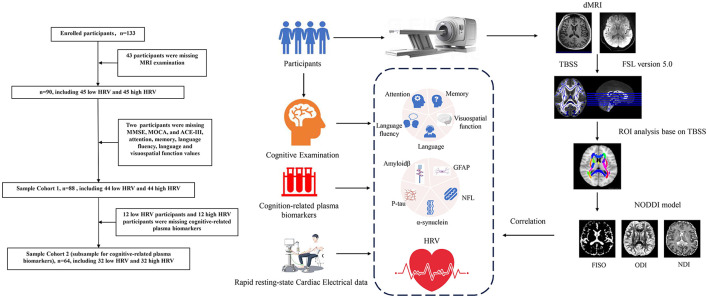
The enrollment process and flowchart of the participant. NODDI, neurite orientation dispersion and density imaging; TBSS, tract-based spatial statistics; HRV, heart rate viability; BPM, beat per minute; RMSSD, the root mean square of successive differences; SDNN, the standard deviation of all NN intervals; SDSD, the standard deviation of the differences between consecutive NN intervals; TP, total power; HF, high frequency; LF, low frequency; VLF, very low frequency; ULF, ultra-low frequency; pNN50, percentage of adjacent intervals that varied by >50 ms; MMSE, mini-mental state examination; MOCA, Montreal Cognitive Assessment; ACE-III, Addenbrooke's Cognitive Examination version III; RCFT-I, Rey Complex Figure Test-Immediate recall; RCFT-D, Rey Complex Figure Test-Delayed recall; CDR, Clinical Dementia Rating; Aβ42, Amyloidβ-42; Aβ40, Amyloidβ-40; GFAP, glial fibrillary acidic protein; NFL, Neurofilament light chain; P-tau, phospho-tau181.

The enrollment process and flowchart are depicted in Figure 1. Participants in this study were consecutively recruited from the Cognitive Psychology Outpatient Department of Fujian Union Hospital from May 2022 to February 2023. Participants voluntarily sought cognitive testing with concerns about their own cognitive functions. The inclusion criteria required that participants have no significant cognitive deficits on standardized tests and clinical assessments. Participants went through a magnetic resonance imaging (MRI) examination on the day they completed the neuropsychological assessment. All participants signed informed consent forms before the MRI examination. Exclusion criteria as follows: other neurological disorders, cognitive impairment, obesity, severe heart disease, arrhythmias, atrial fibrillation, respiratory system diseases, mental illnesses (i.e., schizophrenia or depression), severe endocrine disorders, injuries or surgeries within the past 6 months, use of illegal anesthetics or alcohol abuse, uncorrected vision or hearing, pregnancy or breastfeeding, use of psychotropic drugs, anesthetic analgesics, benzodiazepines, and psychoactive substances were excluded from the study. Additionally, participants were instructed to abstain from caffeine and nicotine for at least 4 h and to avoid alcohol consumption for at least 12 h. Demographic characteristics were collected, including age, sex, education, and Fazekas scales.

### 2.3 Assessment of cognitive function

Global cognition, including Clinical Dementia Rating (CDR), Mini-mental State Examination (MMSE), Montreal Cognitive Assessment (MOCA), Rey Complex Figure Test-Immediate recall (RCFT-I), Rey Complex Figure Test-Delayed recall (RCFT-D) and Addenbrooke's Cognitive Examination version III (ACE-III) scales were conducted by a neurologist with 5 years of experience. Addenbrooke's Cognitive Examination version III (ACE-III) scales were used to assess cognitive function in five subdomains, including attention, memory, language, language fluency and visuospatial function (31).

### 2.4 Determination of plasma biomarkers

Blood samples were collected into EDTA-coated vacuum containers. After centrifugation, blood samples were stored at −80°C and thawed immediately before composition quantification. The levels of plasma amyloid β-42 (Aβ42), amyloid β-40 (Aβ40), glial fibrillary acidic protein (GFAP), neurofilament light chain (NFL), phospho-tau 181 (P-tau) and α-synuclein were measured using the Human Neurology 3-Plex A Assay (N3PA) kit on an automatic single-molecule-array (SIMOA) instrument (Quanterix Corp, MA, USA) according to the manufacturer's protocol.

### 2.5 MRI data acquisition

All the images were acquired using a 3T MR scanner (MAGNETOM Prisma, Siemens Healthcare) with a 64-channel head coil. The foam was placed around the head of each participant before the test to reduce head movement. All participants were asked to refrain from moving their heads during the MRI scan. Diffusion-weighted images were obtained by using a multi-shell echo-planar imaging sequence, which consisted of four *b*-values (0, 1,000, 2,000, and 3,000 s/mm^2^) along 6, 30, 30, and 30 gradient directions, respectively. Seventy-two slices with a thickness of 2 mm were used. The other scan parameters were as follows: repetition time (*T*_R_), 5,800 ms; time to echo (*T*_E_), 91 ms; field-of-view (FOV), 215 × 215 mm^2^; GeneRalized Autocalibrating Partial Parallel Acquisition, 2; slice acceleration factor, 2; number of averages, 1; voxel size, 2 × 2 × 2 mm^3^, without gap; and acquisition time, 9 min 44 s. High-resolution T1-weighted images were acquired using a Magnetization Prepared-Rapid Gradient Echo sequence with the following parameters: *T*_R_, 2,300 ms; *T*_E_, 2.32 ms; FOV, 240 × 240 mm^2^; number of averages, 1; voxel size, 0.9 × 0.9 × 0.9 mm^3^,192 slices; and acquisition time, 4 min 44 s. We used parallel imaging and phase partial fourier (7/8) to accelerate the scanning speed of the MP-RAGE sequence.

### 2.6 Postprocessing of MRI data

All images were scrutinized and any affected by artifacts, noise, or head movement were excluded. The remaining images were processed into raw, preprocessed files with specific indices. (1) The initial DICOM format images were converted into NIFTI files using the MRIcron software package (http://www.nitrc.org/projects/mricron). (2) An average b = 0 s/mm^2^ image from each diffusion-weighted shell served as a reference for subsequent steps. (3) We performed motion and eddy current correction using FSL's eddy tool in the FMRIB's Diffusion Toolbox (FDT). (4) Brain mask generation using a brain extraction tool (BET) and distortion correction via registration of individual T1W and dMRI data. (5) We used visual inspection to avoid severe geometric distortions, ensuring that all images met the quality control criteria before further analysis. (6) The FMRIB's Diffusion Toolbox (FSL-FDT, included in FSL version 5.0 (http://fsl.fmrib.ox.ac.uk/fsl/fslwiki/FDT), was utilized to remove the skull from the images. Following this, the NODDI parameters (including NDI, ODI, and FISO) were calculated with NODDI_toolbox (www.nitrc.org/projects/noddi_toolbox) in MatLab R2022b (The MathWorks Inc, Natick, MA, USA).

### 2.7 TBSS and ROI analysis

Tract-based spatial statistics (TBSS, part of FSL version 5.0, http://fsl.fmrib.ox.ac.uk/fsl/fslwiki/TBSS/UserGuide) was conducted with the FSL software. In our TBSS analysis pipeline, the skeleton projection of NDI, ODI, and FISO were based on the FA skeleton, which is consistent with the standard TBSS method. The main steps were as follows: 1) All individual FA images were non-linearly registered to the FMRIB58_FA template using FNIRT (FMRIB's Non-linear Registration Tool). The FMRIB58_FA template is already in Montreal Neurological Institute (MNI) 152 space standard space.; 2) An average FA image was created from continuous scan images of all subjects in this common space and refined to generate an average WM skeleton representing the center of all fiber bundles with a threshold >0.2; 3) Each subject's FA data is then projected onto a mean white matter skeleton by assigning each skeleton voxel the highest FA value from the local perpendicular tract, ensuring alignment across subjects. The NDI, ODI and FISO images were subjected to the same non-linear deformations at the individual level and projected onto the FA skeleton using nearest-neighbor projection. This ensures that the projection points for all indices are consistent with the voxels of the FA skeleton. Therefore, the values of NDI, ODI, and FISO on the skeleton originated from the same spatial locations, avoiding the situation where projection points for different indices come from different voxels; 4) The FSL was used to conduct double independent sample *t*-test and replacement test between the two groups (replacement times 5,000 times), and the differences in the NDI, ODI and FISO values of the WM between the two groups were detected. For the presentation of differences in specific brain regions, the ROIs from Johns Hopkins University ICBM-DTI-81 White-Matter Labels (32) were used in this analysis. The images metrics for NODDI were transformed to MNI152 space, and then the mean values of FISO, NDI, and ODI were extracted from each tract. Age and sex were added as covariates. The false discovery rate (FDR) was used for multiple-comparison corrections.

### 2.8 Acquisition of rapid resting-state cardiac electrical data

The assessments were conducted in a designated and tranquil room. All participants were mandated to obtain a restful night's sleep of 6 to 8 h before the examination. The data was consistently gathered at the same time in the early morning while participants were fasting. Before the tests, individuals were asked to sit in a relaxed position for a 5-min period. They were instructed to keep their eyes closed, remain conscious, and wear noise-canceling headphones. In the meantime, a 3-min segment of resting-state electrocardiogram (ECG) was simultaneously recorded.

Electrodes for limb leads (aVR, aVL, and aVF) were attached to the patient's wrists and one ankle to capture cardiac electrical activity. The KARDi2/4-B Autonomic Function Mapping ECG system, manufactured by NeuroMed in China, was utilized for both recording and analyzing the subtle millivolt-level fluctuations within the ECG signal. The system automatically identifies and calculates the mean of the three RR intervals preceding and following any normal beat, and applies markers to refine the data by excluding ectopic beats. Incorporating the Pan-Tompkins algorithm integrated within the system, it performed an analysis of time-domain heart rate variability (HRV) indices, such as pNN50, RMSSD, SDNN, and SDSD, as well as frequency-domain indices, which encompass TP, HF, LF, VLF, and ULF. Participants were split in two groups on the basis of HF [median = 111.97 ms^2^; low HRV = 40.99 ms^2^ (median =46.39 ms^2^); high HRV = 235.41 ms^2^ (median = 156.86 ms^2^)] as it has been recommended (4) and extensively used (33, 34).

### 2.9 Statistical analysis

All statistical analyses were performed using SPSS (Version 26.0; Chicago, Illinois, USA). All statistical analyses were defined as a two-sided *p* < 0.05. Data were tested for normal distribution by the Kolmogorov-Smirnov test. Continuous variables were expressed as the mean ± standard deviation by *t*-test. The non-normal distribution variables were expressed as median (interquartile range, IQR) and compared by a non-parametric test. The Chi-squared and Fisher's exact tests were used to compare categorical variables. Finally, Pearson's correlation analysis and partial correlation analysis (controlling for sex, age and total years of education) were utilized to determine associations between abnormal WMs and HRV, and cognitive function. The statistical significance threshold was set at *p* < 0.05.

## 3 Results

### 3.1 Demographic characteristics of participants

Sample cohort 1 included 44 Low HRV, and 44 High HRV participants (Table 1). The table showed no significant differences in sex, education, Fazekas scales, MMSE, CDR, attention, language fluency, and language (*p* > 0.05), but significant differences in age, BPM, RMSSD, SDSD, TP, HF, LF, VLF, ULF, pNN50, MOCA, ACE-III, RCFT-I, RCFT-D, memory, and visuospatial function between the two groups (*p* < 0.01).

**Table 1:** Characteristics of the study participants (sample cohort 1).

**Characteristics**	**Low HRV (*n* = 44)**	**High HRV (*n* = 44)**	**F/Z or χ2 value**	***P*-value**
Age	64.73 ± 11.40	57.32 ± 12.29	0.514	0.004^**^
Female (*n*, %)	19 (43.18%)	28 (63.63%)	3.699	0.054
BPM	73.43 ± 16.88	67.31 ± 8.79	5.509	0.036^*^
Education (years)	9.05 ± 4.63	10.70 ± 5.91	2.192	0.146
Fazekas scale	1.00 (1.00)	0.00 (1.00)	−1.804	0.071
RMSSD (ms)	13.32 ± 5.70	31.10 ± 12.23	11.107	<0.001^***^
SDSD (ms)	12.33 ± 5.70	31.18 ± 12.26	11.199	<0.001^***^
TP (ms^2^)	197.74 (329.04)	764.61 (635.45)	−6.034	<0.001^***^
HF (ms^2^)	40.99 (46.39)	235.41 (156.86)	−8.078	<0.001^***^
LF (ms^2^)	37.50 (67.90)	160.33 (267.06)	−5.333	<0.001^***^
VLF (ms^2^)	33.02 (71.62)	141.46 (126.68)	−4.732	<0.001^***^
ULF (ms^2^)	40.74 (120.42)	166.42 (255.89)	−3.755	<0.001^***^
pNN50 (%)	0.00 (1.20)	4.75 (10.73)	−5.488	<0.001^***^
MMSE	27.50 (6.75)	29.00 (3.75)	−1.935	0.053
MOCA	24.00 (12.00)	26.50 (9.75)	−2.056	0.040^*^
ACE-III	79.00 (35.25)	89.50 (23.75)	−2.234	0.025^*^
RCFT-I	26.00 (17.50)	34.00 (7.38)	−2.751	0.006^**^
RCFT-D	5.00 (8.63)	11.00 (10.25)	−3.205	0.001^***^
CDR	0.50 (0.50)	0.50 (0.50)	−1.212	0.226
Attention	17.00 (4.00)	18.00 (2.00)	−1.824	0.068
Memory	15.66 ± 7.78	19.52 ± 7.52	0.024	0.020^*^
Language fluency	8.07 ± 4.45	9.14 ± 3.17	1.351	0.198
Language	24.00 (6.75)	24.00 (4.75)	−1.430	0.153
Visuospatial function	14.00 (5.75)	15.00 (3.75)	−2.351	0.019^*^

Data were tested for normal distribution by the Kolmogorov-Smirnov test. Continuous variables were expressed as the mean ± standard deviation by t-test. The non-normal distribution variables were expressed as median (interquartile range, IQR) and compared by a non-parametric test.

HRV, heart rate viability; BPM, beat per minute; RMSSD, the root mean square of successive differences; SDNN, the standard deviation of all NN intervals; SDSD, the standard deviation of the differences between consecutive NN intervals; TP, total power; HF, high frequency; LF, low frequency; VLF, very low frequency; ULF, ultra-low frequency; pNN50, percentage of adjacent intervals that varied by >50 ms; MMSE, mini-mental state examination; MOCA, Montreal Cognitive Assessment; ACE-III, Addenbrooke's Cognitive Examination version III; RCFT-I, Rey Complex Figure Test-Immediate recall; RCFT-D, Rey Complex Figure Test-Delayed recall; CDR, Clinical Dementia Rating (sum of boxes).

^***^P < 0.001, ^**^P < 0.01, ^*^P < 0.05.

Sample cohort 2 included 32 low HRV, and 32 high HRV participants, the table showed no significant differences in sex, education, Aβ42, Aβ40, P-tau, GFAP and α-synuclein (*p* > 0.05), but significant differences in age and Aβ42/Aβ40 between the two groups (*p* < 0.05; Table 2).

**Table 2:** Characteristics of the study participants (sample cohort 2).

**Characteristics^a^**	**Low HRV (*n* = 32)**	**High HRV (*n* = 32)**	**F/Z or χ2 value**	***P*-value**
Age	63.94 ± 10.83	55.28 ± 12.00	0.713	0.004^**^
Female (*n*, %)	16 (50.00%)	22 (68.75%)	2.332	0.127
Education (years)	8.28 ± 4.85	10.72 ± 6.20	1.506	0.085
Aβ42	6.59 ± 1.63	6.74 ± 1.79	0.415	0.712
Aβ40	126.92 ± 32.67	115.84 ± 30.17	0.105	0.164
Aβ42/Aβ40	0.05 (0.01)	0.06 (0.02)	−2.259	0.024^*^
P-tau	1.89 (1.53)	2.08 (1.53)	−0.007	0.995
GFAP	90.54 (98.67)	77.26 (69.03)	−0.859	0.390
NFL	21.21 (22.48)	13.85 (13.99)	−1.853	0.064
α-synuclein	13,231.73 (1,7501.36)	12529.79 (1,8145.21)	−0.725	0.468

Data were presented as number (percentage), median (IQR).

HRV, heart rate viability; BPM, beat per minute; Aβ42, Amyloidβ-42; Aβ40, Amyloidβ-40; GFAP, glial fibrillary acidic protein; NFL, Neurofilament light chain; P-tau, phospho-tau 181.

^**^P < 0.01, ^*^P < 0.05.

^a^12 low HRV participants and 12 high HRV participants were missing cognitive-related-plasma biomarkers.

### 3.2 The results of TBSS analysis (sample cohort 1)

TBSS statistical analysis showed significant differences in FISO in several cerebral regions between low HRV and high HRV group, including the left and right anterior corona radiata (ACR), Body of corpus callosum (CCB), Genu of corpus callosum (CCG), Splenium of corpus callosum (CCS), left superior corona radiata (SCR), which were hereinafter referred to as comprehensive abnormal fiber bundles (*p* < 0.05).

**Figure 2:**
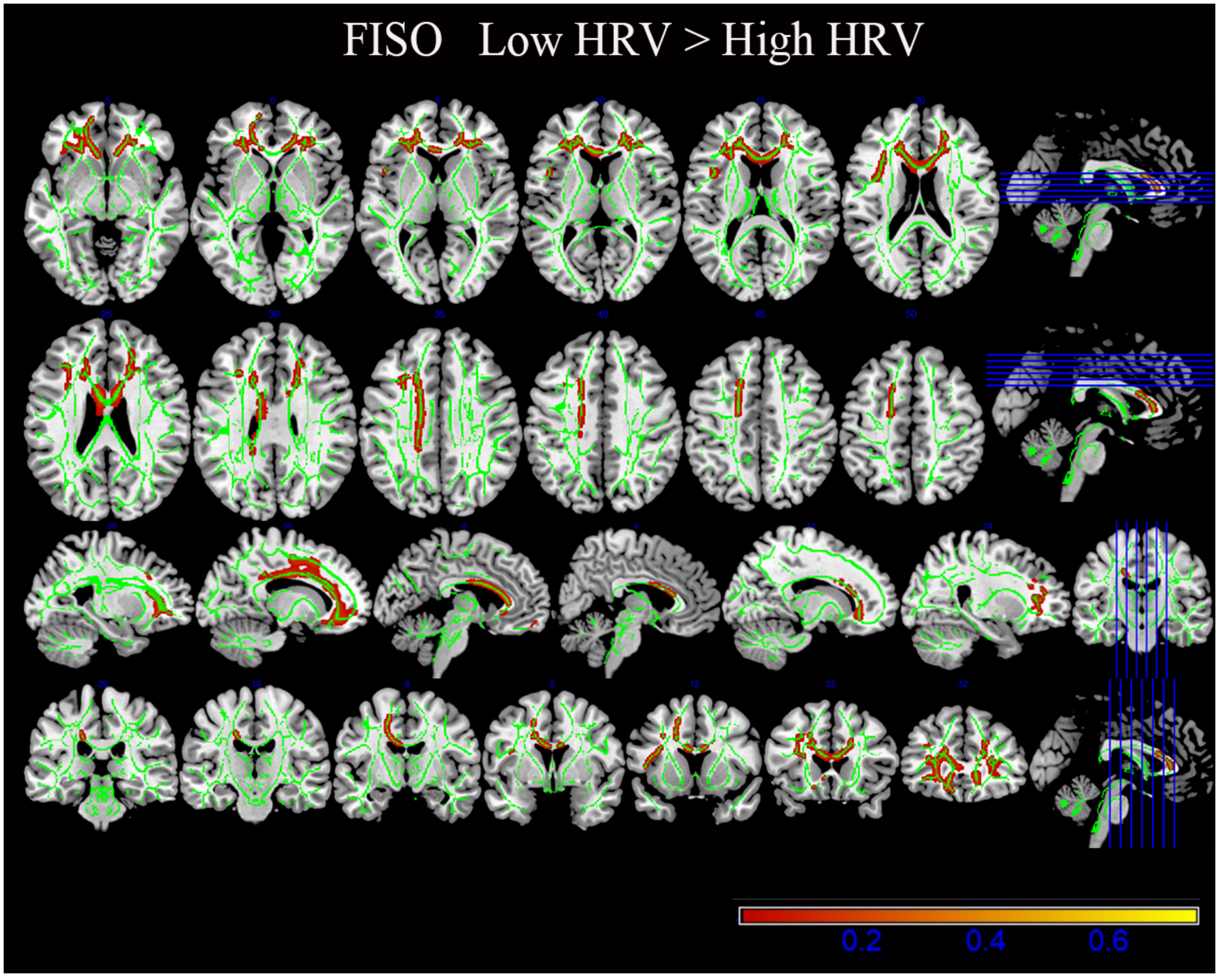
The results of diffusion metrics by tract-based spatial statistics (TBSS). Compared with the High HRV group, the white matter regions of the Low HRV patients showed increased FISO (*P* < 0.05). FISO, isotropic volume fraction; HRV, heart rate viability.

There was no significant difference in the NDI and ODI values between the two groups (*p* > 0.05; Figure 2, Table 3).

**Table 3:** Anatomical regions of tract-based spatial statistics results (High HRV <Low HRV).

**NODDI**	**Cluster index**	**Voxels**	***P***	**Anatomical regions (Top 5)**	**MAX X (mm)**	**MAX Y (mm)**	**MAX Z (mm)**
FISO	2	4,409	0.021	Anterior corona radiata L:26.8893 Body of corpus callosum:14.0598 Genu of corpus callosum:10.8963 Superior corona radiata L:5.2724 Splenium of corpus callosum:0.1757	−26	34	5
	1	1,323	0.035	Anterior corona radiata R:49.4444 Genu of corpus callosum:8.3333 Body of corpus callosum:0.5556	22	41	8

HRV, heart rate variability; FISO, free-water isotropic volume fraction.

### 3.3 ROI analysis results based on TBSS analysis (sample cohort 1)

**Figure 3:**
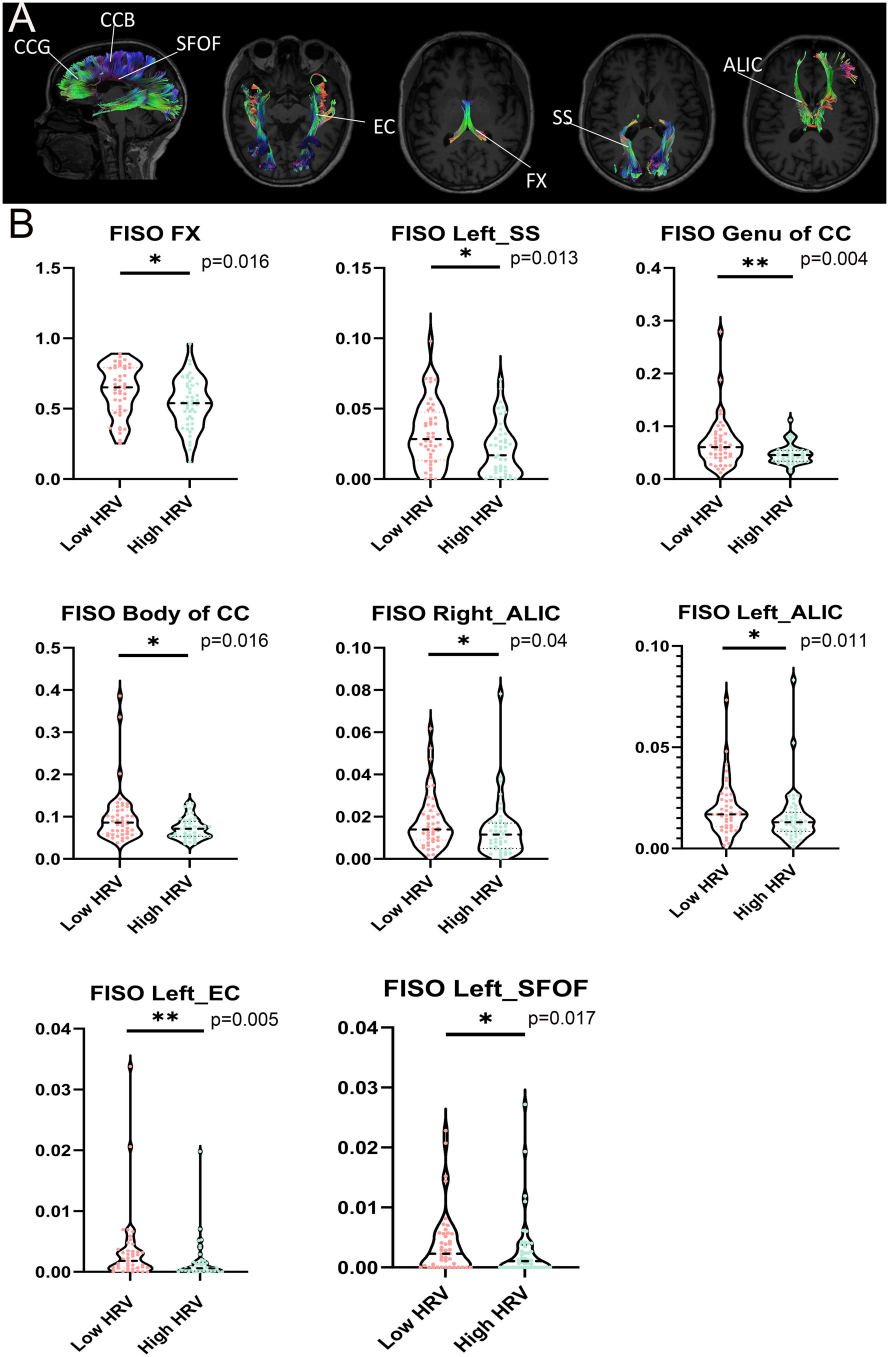
**(A)** Significant tracts from tract-of-interest analysis comparing between low HRV and High HRV. Tracts obtained using the JHU White Matter (WM) Tractography Atlas. **(B)** Violin plots show the important fiber bundles of FISO in the region of interest (ROI) by cluster-based spatial statistics (TBSS). False discovery rate (FDR) correction was performed for multiple comparisons, with statistical significance of *P* < 0.05. HRV, heart rate viability; CCB, Body of corpus callosum; CCG, Genu of corpus callosum; FX, fornix; SS, sagittal stratum; ALIC, anterior limb of internal capsule; EC, external capsule; SFOF, left superior fronto-occipital fasciculus. ^**^P < 0.01, ^*^P < 0.05.

Compared with the High HRV group, FISOs of CCB, CCG, fornix (FX), left sagittal stratum (SS), bilateral anterior limb of internal capsule (ALIC), left external capsule (EC) and left superior fronto-occipital fasciculus (SFOF) in the low HRV group increased to different degrees; (*p* < 0.05; Figure 3). In addition, no significant differences in other fiber bundle indicators were observed between the groups (*p* > 0.05).

### 3.4 Association of aberrant NODDI indicators with cognition (sample cohort 1)

**Figure 4:**
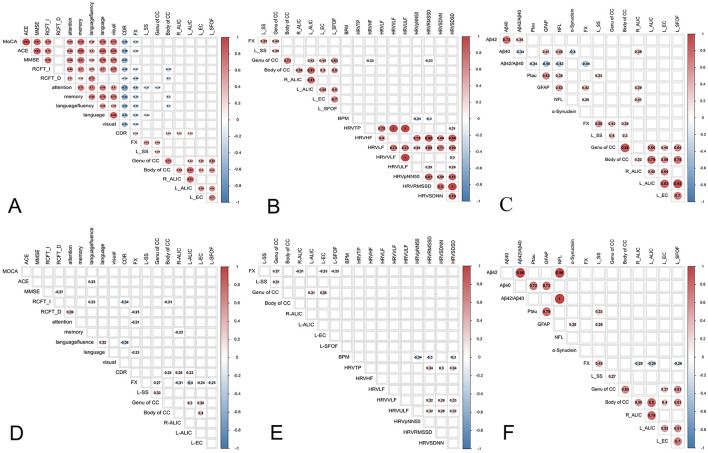
The results of pearson's correlation analysis **(a–c)** and partial correlation analysis **(d–f)** between abnormal WMs with cognitive function, heart rate viability (HRV) and cognition-related biomarkers. **(a)** Correlation between the abnormal fiber bundles and cognitive function. **(b)** Correlation between the abnormal fiber bundles and HRV. **(c)** Correlation between abnormal fiber bundles and cognition-related plasma biomarkers. **(d)** Partial correlation analysis between abnormal fiber bundles and cognition-related plasma biomarkers. **(e)** Partial correlation analysis between abnormal fiber bundles and HRV. **(f)** Partial correlation analysis between abnormal fiber bundles and cognition-related plasma biomarkers. HRV, heart rate viability; BPM, beat per minute; RMSSD, the root mean square of successive differences; SDNN, the standard deviation of all NN intervals; SDSD, the standard deviation of the differences between consecutive NN intervals; TP, total power; HF, high frequency; LF, low frequency, VLF, very low frequency; ULF, ultra-low frequency; pNN50, percentage of adjacent intervals that varied by >50 ms; MMSE, mini-mental state examination; MOCA, Montreal Cognitive Assessment; ACE-III, Addenbrooke's Cognitive Examination version III; RCFT-I, Rey Complex Figure Test-Immediate recall; RCFT-D, Rey Complex Figure Test-Delayed recall; CDR, Clinical Dementia Rating; Aβ42, Amyloidβ-42; Aβ40, Amyloidβ-40; GFAP, glial fibrillary acidic protein; NFL, Neurofilament light chain; P-tau, phospho-tau 181; CCB, Body of corpus callosum; CCG, Genu of corpus callosum; FX, fornix; SS, sagittal stratum; ALIC, anterior limb of internal capsule; EC, external capsule, SFOF, left superior fronto-occipital fasciculus.

Pearson's correlation analysis showed that abnormalities in the WM microstructure were closely associated with a wide range of cognitive domains (*p* < 0.05; Figure 4a).

Partial correlation analysis revealed that the FISO of CCB, right ALIC and left ALIC were positively associated with CDR, the FISO of FX was associated with RCFT-D, attention and language, the FISO of CCB was negatively associated with RCFT-I, the FISO of right ALIC were negatively associated with memory (*p* < 0.05; Figure 4d). Furthermore, no association was found between other fiber bundle indexes and cognitive function (*p* > 0.05; Figure 4d).

### 3.5 Association of aberrant NODDI indicators with HRV (sample cohort 1)

Pearson's correlation analysis showed that the FISO of CCG were negatively associated with RMSSD and SDSD (*p* < 0.05; Figure 4b).

Partial correlation analysis revealed that no association was found between fiber bundle indexes and HRV (*p* > 0.05; Figure 4e).

### 3.6 Association of aberrant NODDI indicators with cognition-related plasma biomarkers (sample cohort 2)

Pearson's correlation analysis found that the FISO of FX was negatively associated with Aβ42/Aβ40 and positively associated with NFL, GFAP and p-tau 181.The FISO of left SS and CCB were positively associated with p-tau 181. The FISO of right ALIC was positively associated with NFL, GFAP and p-tau 181 (*p* < 0.05; Figure 4c).

Partial correlation analysis revealed that the FISO of FX was positively associated with GFAP. The FISO of left SS and CCG were positively associated with p-tau 181 (*p* < 0.05; Figure 4f). The scatter plot was used to display the correlation between Fractional Isotropic Volume Fraction (FISO) and cognitive function, as well as cognition-related plasma biomarkers (Supplementary Figure S1).

## 4 Discussion

In this study, we utilized NODDI to analyze variations in the integrity and microstructure of WM in participants with low HRV and high HRV. We found that participants in the low HRV group exhibited WM discrepancies in various regions and severities, and these microstructure changes were associated with cognitive function, cognition-related plasma biomarkers to varying degrees. Taken together, these findings indicate that microstructural abnormalities in WM may be indicative of underlying histopathological changes linked to cognitive decline. These findings may provide novel insight into the effects of HRV on cognitive domains.

NODDI, as an innovative diffusion MRI approach, has the three most commonly derived NODDI metrics (ODI, NDI, and FISO). FISO reflects the content of free water in tissue, which is used to quantify the volume of voxels occupied by free-flowing cerebrospinal fluid (35). Cumulative studies have shown that the increase in FISO may indicate a decrease in the microstructural integrity of the WM (29, 36, 37). In this study, we found several microstructural anomalies in FISO, which implies axonal loss in the WM is the primary pathological change in the low HRV group. Indeed, the potential white matter tract injury may begin with axonal loss (38). Besides, the elevated FISO levels suggest an expansion of the extracellular fluid volume, a condition typically associated with neuroinflammatory (39) which has been suggested to contribute to neurodegenerative diseases and cognitive dysfunction (40).

Notably, TBSS and ROI analysis showed that the abnormal WM integrity was mainly manifested in the anterior regions of the brain and corpus callosum (CC), mainly linked to the frontal and corpus callosum. Our findings seem to support this theory of the frontal-corpus callosum pathways. The frontal region is a complex brain region through which a variety of WM fibers pass, mainly connecting and associative fibers. Multiple previous studies have confirmed that the front of the brain is related to attention, memory, motion, language, executive function (41). Damage or dysfunction of the frontal or corpus callosum may lead to impairment of any one or several of the aforementioned functions, which could significantly affect an individual's daily life and social functioning (41, 42). ROI analysis also found that the FISO values of FX, CCB, right ALIC and left ALIC were associated with the cognitive function and broad cognitive domains to varying degrees, which is consistent with the previous findings (43, 44). It seems that low HRV are prone to affect the WM in the anterior regions of the brain. This can be explained by several potential mechanisms. Changes in the integrity of the fibers (right and left ALIC, CC) in the regions associated with autonomic nervous function, such as the corpus callosum and basal ganglia (45), further support the basic theory that HRV is closely linked to the dysfunction of the autonomic regulatory regions. The frontal cortex is also the main region of autonomic regulation (46). ALIC is an important white matter structure in the brain that carries nerve fibers from the cerebral cortex to the brain stem. The brainstem is the key part of autonomic regulation (45). EC and SFOF are involved in connecting the cerebral cortex to deeper brain structures, including those involved in autonomic nervous system (ANS). Therefore, we speculate that the damage to WM in low HRV patients might result from the injury of ANS. In addition, despite a lack of consensus, cumulative studies have shown that low HRV were associated with levels of brain-derived neurotrophic factor (BDNF) in the frontal cortex (47, 48). BDNF is a widely distributed protein in the central nervous system that plays a critical role in the growth, survival, and differentiation of neurons (48). HRV could influence BDNF concentrations within the frontal cortex, potentially causing degeneration in white matter.

Although the microstructural changes detected by NODDI could not directly prove low HRV with cognitive impairments, our study results showed that abnormal fiber bundles in WM of low HRV group were associated with cognitive-related regions. This damage may lead to decreased efficiency in information transfer, thereby affecting the normal functioning of these cognitive abilities. Previous studies had also confirmed that WM damage in cognitive-related regions is closely related to the decline in executive function, memory, and attention, such as the corpus callosum, fornix, and internal capsule. A host of studies have documented the association of FX with long-term memory and spatial memory (49), that of SS with visual memory and spatial cognition (50), the role of SFOF in visual processing and attention (51), the role of CCB and CCG in attention, memory, and emotional processing (52), and that of EC and ALIC in memory and executive function (53). In a similar line, the damaged WM areas were also associated with neurological diseases which also supports that low HRV contributes to the occurrence of cognitive diseases (54, 55). Furthermore, most of the cognitive assessments showed significant differences between groups in our study (Table 1), which also confirmed the trend of cognitive decline in individuals with low HRV. This is similar to the findings of a recent study (56). Although MMSE, attention, and language did not show significant differences between groups, Cohen's d measurements (Supplementary Table S1) revealed small to medium effect sizes, indicating a trend toward cognitive decline. Additionally, WM abnormalities in FISO were only detected between groups, and FISO is known to be sensitive to early WM damage (57). We speculate that individuals with low HRV may be in the early stages of cognitive decline.

Our study also found a significant difference in the Aβ42/Aβ40 ratio between the Low HRV group and the High HRV group. The decreased Aβ42/Aβ40 ratio may indicate that individuals in the low HRV group are more susceptible to cognitive impairment. The mechanism may be related to neuroinflammation and vascular regulation. First, amyloid beta may be associated with neuroinflammation (58) and the changes in both amyloid beta and neuroinflammation tend to happen relatively early in the process, perhaps even before signs of cognitive impairment (59). We speculate that low HRV may be associated with higher inflammatory markers or may have a role in decreased clearance of Aβ42. Second, autonomic nervous system (ANS) imbalance caused by reduced HRV can exacerbate neuroinflammatory responses. Dong-Hun Lee found that the ANS and the hypothalamic-pituitary-adrenal (HPA) axis can induce excessive inflammatory responses in the central nervous system (CNS), leading to neuroinflammation (60), a finding also confirmed by Aleksandar Sic's study (61). HRV, an indicator of autonomic nervous system regulation of cardiovascular function, might affect cerebral blood flow and blood-brain barrier function, reducing the clearance efficiency of Aβ (62) and accelerating the deposition of Aβ42 in the vascular walls, which further lowers the Aβ42/Aβ40 ratio.

Although the intergroup difference of NFL did not reach the threshold of statistical significance, its trend is consistent with other indicators of neurodegeneration and cognitive decline. Reduced HRV may exacerbate neuronal stress through chronic inflammation or vascular damage (63), leading to increased release of NFL (64). Additionally, studies have found that abnormal Aβ metabolism can further lead to increased NFL release by promoting neuronal damage (65). We speculate that HRV may establish a “double-hit” mechanism on AD pathology: simultaneously promoting amyloid toxicity (decreased Aβ42/Aβ40) and neurodegeneration (increased NFL).

Strengths of our study included the recruitment of subjects with complete clinical, neuropsychological data, and plasma biomarker data, which were integrated with DWI data. In addition, for the first time, our study applied NODDI, which facilitates an accurate assessment of the WM integrity in low HRV patients. We also explain the mechanism by which HRV leads to cognitive decline from the perspective of the integrity of WM.

Our study was not without limitations. First, the association between WM integrity and cognition-related plasma biomarkers was investigated in a small sample. Second, the results were derived from Chinese population and caution needs to be taken when they are generalized to other populations. Third, owing to its cross-sectional design, selection bias was inevitable. Fourth, the MNI152 template is mostly applied to young individuals. Although the subjects in this study were older, we employed rigorous visual inspection and eddy current correction to avoid misregistration. In future work, we will attempt to use more appropriate templates for our population (such as “most representative subject” in https://fsl.fmrib.ox.ac.uk/fsl/docs/#/diffusion/tbss?id=user-guide or study-specific templates in https://dti-tk.sourceforge.net/pmwiki/pmwiki.php?n=Documentation). Fifth, EPI-induced geometric distortion correction was not applied due to the lack of available field maps or reverse phase encoding acquisitions. However, we performed visual inspection of all diffusion images to ensure that no severe distortions were present, and all images met the quality control criteria. Future studies could benefit from incorporating EPI distortion correction techniques, to further improve the accuracy of white matter microstructural analysis. Sixth, considering that white matter hyperintensities (WMH) may affect the measured NODDI metrics, we will further investigate WMH volume in future work to better understand these differences.

In conclusion, our study evidences that the microstructural changes of WM in Low HRV are associated with cognitive domains and cognition-related plasma biomarkers. Low HRV may lead to worse cognitive performance through the damage of the genu of the corpus callosum pathway.

## Data Availability

The original contributions presented in the study are included in the article/Supplementary material, further inquiries can be directed to the corresponding author.

## Supplementary material

Supplementary Figure S1 The correlation between FISO and cognitive function, as well as cognition-related plasma biomarkers. FISO, fractional isotropy; RCFT-I, Rey Complex Figure Test-Immediate recall; RCFT-D, Rey Complex Figure Test-Delayed recall; CDR, Clinical Dementia Rating global rating; GFAP, glial fibrillary acidic protein; P-tau, phospho-tau 181.

Supplementary Table S1 The Cohen's d of cognitive assessments between groups. Cohen's d is a standardized measure used to quantify the difference in means between two groups and is commonly employed to assess effect size. Specifically, *d* = 0.2 indicates a small effect, *d* = 0.5 indicates a medium effect, and *d* = 0.8 indicates a large effect. The formula for calculating Cohen's d: Cohen's *d* = $\frac{M_{1}-M_{2}}{S_{pooled}}$ M_1_ and M_2_ are the means of the two groups of data; S_pooled_ is the pooled standard deviation. $S_{pooled}=\sqrt{\frac{\left(N_{1}-1\right)\times SD_{1}^{2}+\left(N_{2}-1\right)\times SD_{2}^{2}}{N_{1}+N_{2}-2}}$. N_1_ and N_2_ are the sample sizes of the two groups of data; SD_1_ and SD_2_ are the standard deviations of the two groups of data.
