# Supplementary figures and images for: Cognitive implications of white matter microstructural changes in individuals with low heart rate variability: a NODDI study
Source: Front Neurol. 2025 May 9;16:1503599. doi: 10.3389/fneur.2025.1503599 (PMC12098083; doi:10.3389/fneur.2025.1503599)

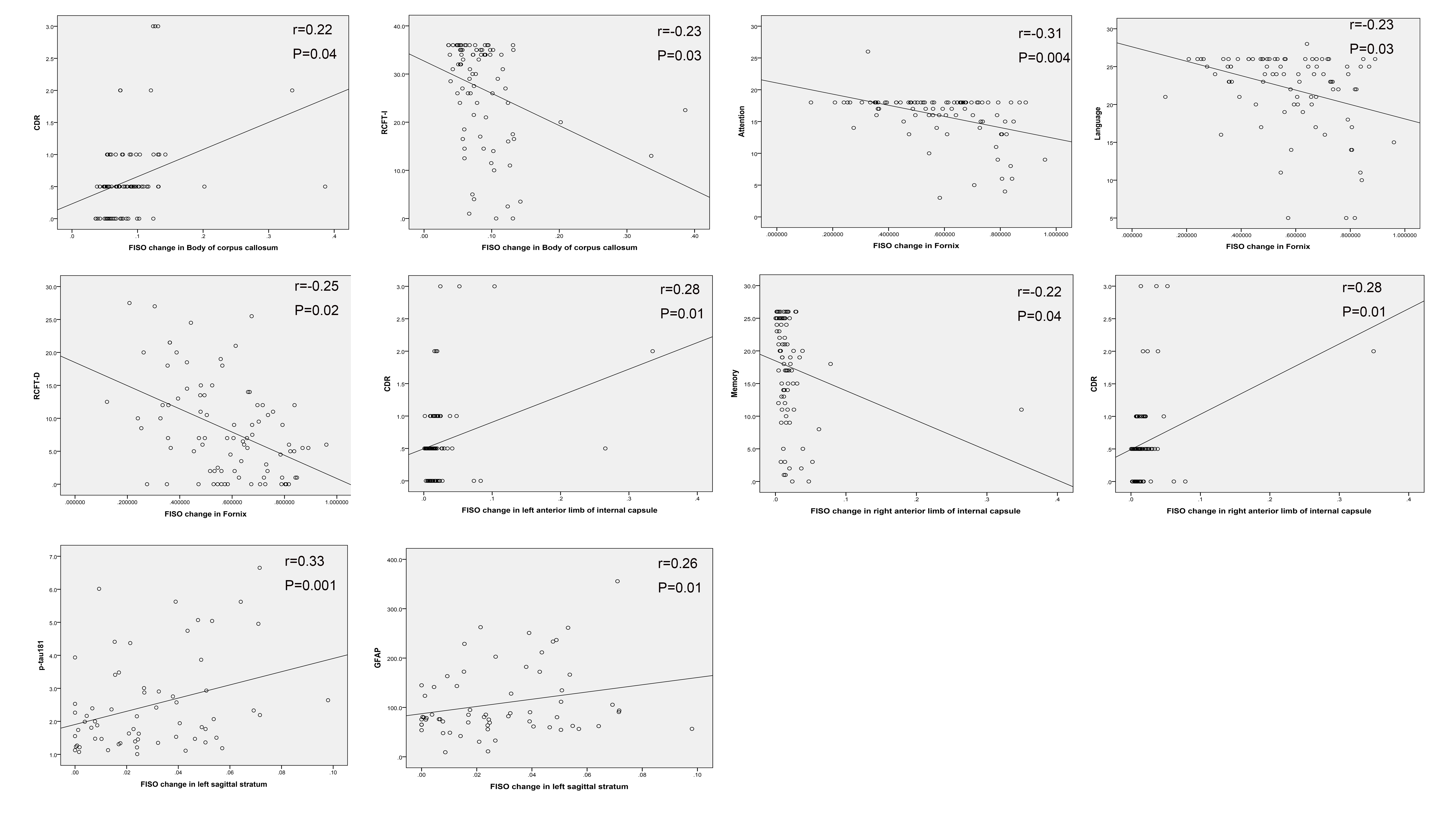

Supplement: Supplementary Figure S1 — The correlation between FISO and cognitive function, as well as cognition-related plasma biomarkers. FISO, fractional isotropy; RCFT-I, Rey Complex Figure Test-Immediate recall; RCFT-D, Rey Complex Figure Test-Delayed recall; CDR, Clinical Dementia Rating global rating; GFAP, glial fibrillary acidic protein; P-tau, phospho-tau 181. [file Image_1.tif]
