# Supplementary material for: Cognitive implications of white matter microstructural changes in individuals with low heart rate variability: a NODDI study
Source: Front Neurol. 2025 May 9;16:1503599. doi: 10.3389/fneur.2025.1503599 (PMC12098083; doi:10.3389/fneur.2025.1503599)
Supplement: Supplementary Table S1 — The Cohen's d of cognitive assessments between groups. Cohen's d is a standardized measure used to quantify the difference in means between two groups and is commonly employed to assess effect size. Specifically, d = 0.2 indicates a small effect, d = 0.5 indicates a medium effect, and d = 0.8 indicates a large effect. The formula for calculating Cohen's d: Cohen's d = M1-M2Spooled M1 and M2 are the means of the two groups of data; Spooled is the pooled standard deviation. Spooled=(N1-1)×SD12+(N2-1)×SD22N1+N2-2. N1 and N2 are the sample sizes of the two groups of data; SD1 and SD2 are the standard deviations of the two groups of data. [file Table_1.docx]

Table S1 The Cohen’s d of cognitive assessments between groups

| Cognitive assessments | Cohen’s d |
| --- | --- |
| MMSE | 0.319 |
| Attention | 0.127 |

Note: Cohen’s d is a standardized measure used to quantify the difference in means between two groups and is commonly employed to assess effect size. Specifically, d = 0.2 indicates a small effect, d = 0.5 indicates a medium effect, and d = 0.8 indicates a large effect.

The formula for calculating Cohen’s d:
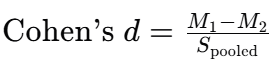


M_1_ and M_2_ are the means of the two groups of data; S_pooled_ is the pooled standard deviation.


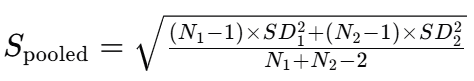


N_1_ and N_2_ are the sample sizes of the two groups of data; SD_1_ and SD_2_ are the standard deviations of the two groups of data.
